# The potential of optical coherence tomography angiography in progressive multiple sclerosis

**DOI:** 10.1007/s00415-026-13659-7

**Published:** 2026-02-07

**Authors:** Jonathan A. Gernert, Hanna Zausinger, Luca Diedrich, Rebecca Wicklein, Linus Kreitner, Tania Kümpfel, Joachim Havla

**Affiliations:** 1 Institute of Clinical Neuroimmunology, LMU Hospital, Ludwig-Maximilians-Universität München, 81377 Munich, Germany; 2 Biomedical Center and University Hospital, Ludwig-Maximilians-Universität München, 82152 Planegg-Martinsried, Germany; 3 Department of Neurology, TUM University Hospital, TUM School of Medicine and Health, Technical University of Munich, Munich, Germany; 4 Chair for AI in Healthcare and Medicine, Technical University of Munich (TUM) and TUM University Hospital, Munich, Germany; 5 Munich Cluster for Systems Neurology (SyNergy), Munich, Germany

**Keywords:** Optical coherence tomography angiography, Retinal vasculature, Progressive multiple sclerosis

## Abstract

Early detection and monitoring of neurodegenerative processes in persons with multiple sclerosis (PwMS) is currently a major challenge. Optical coherence tomography angiography (OCTA) is an emerging method to visualize retinal vascular architecture. However, its use has mainly been investigated in relapsing MS. We evaluated OCTA as a possible complementary method in progressive MS (PMS) in a monocentric, retrospective, cross-sectional study. Eyes with evidence of optic neuritis were excluded from analysis. OCTA images acquired using a Spectralis OCT (Heidelberg Engineering) were analyzed with an established deep learning-based segmentation algorithm. After rigorous quality control, 85 eyes of 62 PwPMS were compared with 64 eyes of 43 age and gender-matched healthy controls (HC). The vessel density in the superficial vascular complex (VD_SVC_ (%)) was reduced in PMS compared to HC (*p* = 0.018). VD_SVC_ correlated negatively with age in PwPMS and HC. Using a Johnson–Neyman analysis, we identified that the disease duration influences the VD_SVC_ in PMS individuals < 57.5 years of age. PwPMS with disease duration > 10 years had reduced VD_SVC_ compared to subjects with ≤ 5 years of disease duration (*p* = 0.049) (corrected for age). Clinical disability (EDSS) negatively correlated with VD_SVC_ in PwPMS (*β* = −0.487, *p* = 0.010). These results suggest that OCTA might be suitable to detect retinal vascular changes in PwPMS. One consequence could be structured and harmonized OCTA investigations as part of routine clinical practice. External validation and longitudinal studies are necessary to further elaborate OCTA´s potential in monitoring PwPMS.

**Supplementary Information:**

The online version contains supplementary material available at 10.1007/s00415-026-13659-7.

## Introduction

Current evidence points to a continuum of inflammation, neurodegeneration and compensatory processes in multiple sclerosis (MS) [1]. However, the progressive disease course poses major clinical challenges due to its complex and not yet sufficiently understood pathophysiology [1]. Therapeutic options for progressive MS (PMS) remain limited and are often only modestly effective in altering disease trajectory [2]. Moreover, a variety of serum [3] and cerebrospinal fluid [4] biomarkers as well as imaging markers [5, 6] have been described. However, there is a lack of reliable, cost-effective and low-risk parameters for early detection and monitoring of disease progression in routine clinical practice. Establishment of valid and easily accessible markers in PMS, therefore, represents an unmet need.

Optical coherence tomography (OCT) has been increasingly used in persons with PMS as it is a non-invasive, reproducible, cost-effective and high-resolution technique to visualize retinal structures that may mirror central nervous system pathologies [7]. Rapid atrophy rates of retinal layers in PMS—explicitly the macular inner as well as the outer nuclear layer (mINL and mONL)—have been reported as parameters of neuroaxonal loss [8]. In addition, longitudinal measurements of the thickness of the macular inner plexiform layer (mIPL) were described as an interesting method to detect early disease progression and synaptic injury [9]. However, a considerable variability has been described in longitudinal measurements of retinal layer thickness, which limits the use of the OCT-derived retinal layer atrophy rates [10].

Over the last decade, optical coherence tomography angiography (OCTA) has emerged as a non-invasive method for imaging the microvasculature of the retina and choroid. This raises the question of whether (OCTA) could be an alternative method for detecting and monitoring microvascular networks in the retina as a measure of disease progression. Its use has been investigated in relapsing MS (RMS) in particular: a meta-analysis reported on reduced vascular density (VD) in the superficial vascular complex (VD_SVC_) in MS subjects across different OCTA devices [11]. In RMS, it has been shown that retinal vessel rarefication is associated with cerebral atrophy, as well as with clinical disability [12, 13]. In addition, further studies evaluated the use of OCTA, for example, in Parkinson's disease or Alzheimer's disease, as a promising tool to assess neurodegenerative processes [14, 15].

In this study, we aimed to investigate whether OCTA holds potential to detect and monitor neurodegenerative processes in a larger cohort of persons with PMS (PwPMS).

## Methods

### Study cohort

Monocentric, retrospective study using patient data from the outpatient clinic of the Institute of Clinical Neuroimmunology, LMU Hospital. We included persons with primary (PPMS) and secondary progressive MS (SPMS) according to [16]. The Expanded Disability Status Scale (EDSS) is reported for scoring disability. Healthy controls (HC) without anamnestic history of neurological or ophthalmological diseases were used as reference cohort. Subjects < 18 years, with >  + 5dpt, < -5dpt, diabetes mellitus, untreated arterial hypertension and ophthalmologic diseases were excluded. Eyes with a clinical history of optic neuritis or with a relevant inter-eye difference in pRNFL (peripapillary retinal nerve fibre layer; absolute (≥ 5 μm) or relative (≥ 5%) difference) and mGCIP (combined macular ganglion cell and inner plexiform layer; absolute (≥ 4 μm) or relative (≥ 4%) difference) were excluded [17], as this focal inflammation is known to result in changes in the retinal vascular network [18] and we focused on the role of OCTA in MS-associated neurodegenerative processes. The local ethics committee waved approval for this study (22–0692). This analysis was conducted according to the Declaration of Helsinki.

### Optical coherence tomography (OCT) and OCT–angiography (OCTA) imaging

OCT and OCTA scans during 07/2021–09/2024 using a Spectralis SD–OCT, Heidelberg Engineering, Heidelberg, Germany, OCT2-Module were screened for inclusion criteria. A ring scan of the optic nerve head was performed to measure the pRNFL thickness, as well as a macular scan to determine the volumes of the macular RNFL (mRNFL), the mGCIP, the mIPL, the mINL and the mOPL (outer plexiform layer). All retinal layers were segmented semi-automatically using the proprietary software provided by the SD–OCT manufacturer (Heidelberg Eye Explorer (Heyex) v2.5.5) and manually corrected if necessary. The scans were checked for sufficient quality by experienced raters according to the OSCAR–IB criteria [19]. OCTA scans were obtained within a 2.9 × 2.9 mm region centered on the fovea centralis. For OCTA images, a rigorous quality control was performed by two independent experts blinded to demographical and clinical data according to the recently proposed OSCAR–MP consensus criteria [19]. For analysis of VD (in %) and the foveal avascular zone (FAZ in mm^2^), we used an updated version of a deep learning-based segmentation tool, trained on a large data set of synthetic OCTA images [20, 21]. This tool is less affected by image artifacts compared to traditional computer vision algorithms and has been reported to outperform established analysis tools [20, 21]. VD of the superficial (VD_SVC_)—extending from the internal limiting membrane (ILM) to the mIPL—and deep vascular complex (VD_DVC_)—spanning from the mIPL to the outer boundary of the mOPL—were analyzed. The VD_SVC_ was further stratified differentiating between vessel diameters of < 10, 10–20 and > 20 µm.

### Statistical analysis

R, version 4.4.1 (RStudio Teams (2024) Posit Software, PBC, Boston, MA) was used for statistical analysis and creation of figures. If two eyes of a person were included, the mean values were used for further analysis. Data are presented as mean with standard deviation (SD) or median with interquartile range (IQR). Inter-rater reliability is shown as Cohen´s kappa coefficient. The chi-square test was used to compare nominally distributed characteristics. Continuous, parametric data were compared using a *t* test, respectively, for nonparametric data the Mann–Whitney *U* test. Linear regression models were used to associate OCTA with OCT, demographic and clinical markers. Multiple linear regression models with interaction terms were used to assess whether an association between clinical and OCTA parameters is influenced by demographic factors. If that was the case, a Johnson–Neyman analysis was performed (to determine the effect modification). After subdividing the PMS cohort based on its disease duration, a rank-based linear regression model was performed considering age as a covariate. Statistical significance was set at *p* values < 0.05.

## Results

### Composition of the study cohort

**Fig. 1:**
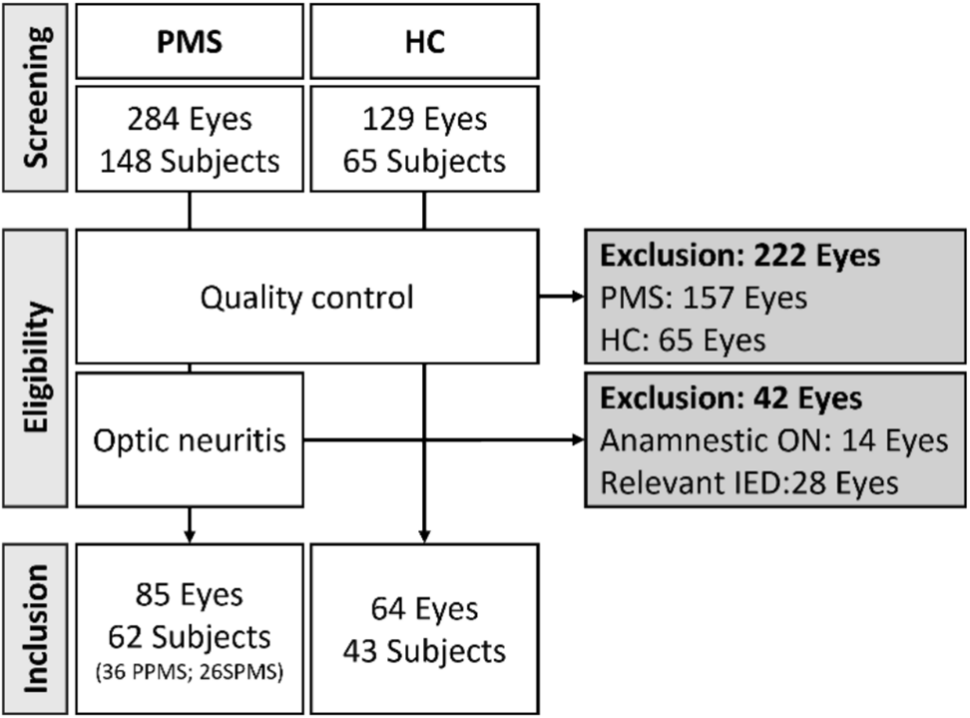
Flow diagram of eyes included in further analysis. *HC* healthy controls, *IED* inter-eye difference (pRNFL, mGCIP; please refer to the *Methods*), *ON* optic neuritis, *PMS* progressive multiple sclerosis, *PPMS* primary progressive multiple sclerosis, *SPMS* secondary progressive multiple sclerosis

In total, we screened OCTA images from 284 eyes of 148 PwPMS. From this cohort, 157 eyes (55%) did not meet the OSCAR–MP quality criteria. Of the remaining 127 eyes, 14 eyes were excluded due to anamnestic optic neuritis and 28 eyes due to significant inter-eye difference of pRNFL and mGCIP. A total of 85 eyes were included in the final analysis (62 PwPMS: 36 PPMS and 26 SPMS) (Fig. 1). From 129 eyes (65 subjects) of an age- and gender-matched HC cohort, 64 eyes (50%) fulfilled the OCTA quality check (Fig. 1). The inter-rater agreement for the screening process to select eligible OCTA images was $\kappa=0.82$. After this quality control, the PMS and HC cohorts included in the further analysis did not differ in terms of age (*p* = 0.089) and gender distribution (*p* = 0.439) (Table 1).

**Table 1:** Characterization of PMS and HC cohorts

	PMS	HC	*p* value
Demographic data			
Eyes	85	64	
Persons	62	43	
Gender (female:male)	33:29	27:16	0.439^a^
Age (years)	53.5 ± 9.48	49.1 ± 15.0	0.089^b^
Clinical data			
Disease duration (years)	10.5 (6–15.8)		
EDSS	4.5 (3–6)		
OCT data			
pRNFL ($\upmu\mathrm{m}$)	**94.5 (87.6–97.9)**	**99.0 (90.2–106.0)**	**0.024**^**c**^
mRNFL (mm^3^)	**0.85 ± 0.11**	**0.92 ± 0.10**	** < 0.001**^**b**^
mGCIP (mm^3^)	**1.87 ± 0.18**	**2.01 ± 0.13**	** < 0.001**^**b**^
mINL (mm^3^)	0.98 ± 0.07	0.97 ± 0.07	0.472^b^
OCTA data			
VD_SVC_ (%)	**35.4 ± 2.4**	**36.6 ± 2.3**	**0.016**^**b**^
VD_SVC_ < 10 μm diameter (%)	10.2 ± 1.1	10.4 ± 1.1	0.368^b^
VD_SVC_ 10–20 μm diameter (%)	18.7 ± 1.6	19.1 ± 1.5	0.243^b^
VD_SVC_ > 20 μm diameter (%)	**6.4 ± 1.0**	**7.0 ± 0.9**	**0.002**^**b**^
VD_DVC_ (%)	43.7 ± 1.5	43.9 ± 1.4	0.328^b^
FAZ area (mm^2^)	0.12 (0.09–0.14)	0.11 (0.09–0.13)	0.440^c^

*DVC* deep vascular complex, *EDSS* expanded disability status scale, *FAZ* fovea avascular zone, *HC* healthy control, *mGCIP* combined macular ganglion cell and inner plexiform layer, *mINL* macular inner nuclear layer, *mRNFL* macular retinal nerve fibre layer, *OCT* optical coherence tomography, *OCTA* optical coherence tomography angiography, *PMS* progressive multiple sclerosis, *pRNFL* peripapillary retinal nerve fibre layer, *SVC* superficial vascular complex, *VD* vessel density

^a^ Chi-square test

^b^ Two-sided *t* test

^c^ Mann–Whitney *U* test

### Comparison of OCT and OCTA between PMS and HC cohort

**Fig.2:**
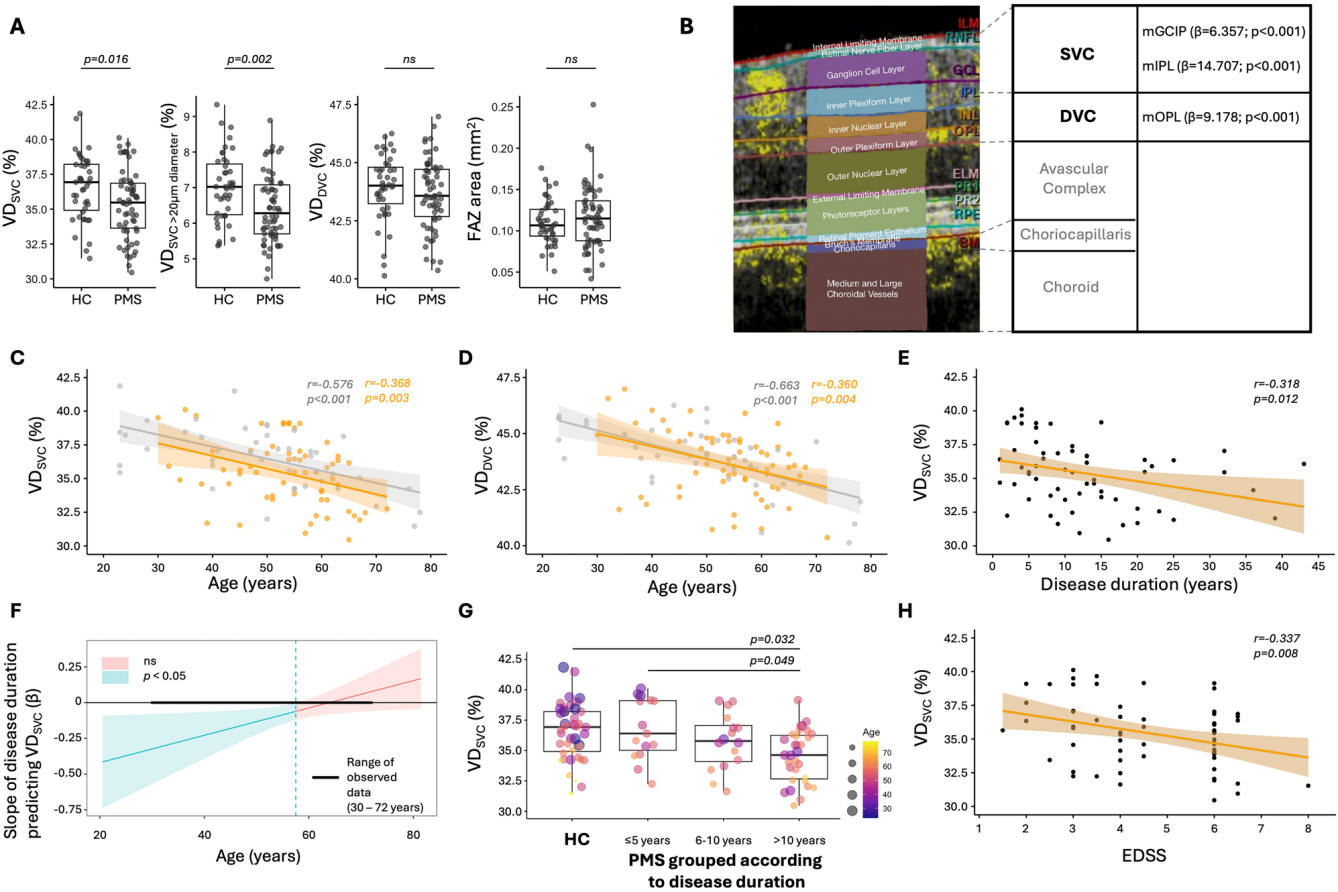
Optical coherence tomography angiography in progressive multiple sclerosis. **A** VD_SVC_ (in %) is decreased in PMS compared to HC. After stratifying for vessel diameter, especially the vessels with > 20µm diameter are reduced in PMS compared to HC. The VD_DVC_ as well as the FAZ area (mm^2^) did not differ between PMS and HC. **B** Image on the left side depicts an OCTA image with retinal layer segmentation of the manufacture’s segmentation algorithm. Within a Heidelberg Spectralis SD–OCT, the SVC ranges from the internal limiting membrane (IML) to the mid of the inner plexiform layer (IPL). The DVC encompasses the lower parts of the IPL as well as the inner nuclear (INL) and outer plexiform layer (OPL). Values of linear regression models for VD_SVC_, VD_DVC_ and corresponding layer volumes are shown on the right side. **C, D** Age negatively correlates with the VD_SVC_ as well as the VD_DVC_. The respective 95% confidence interval (CI) is shown alongside the trend line for the two cohorts PMS (orange) and HC (grey). **E** Disease duration and VD_SVC_ are negatively associated (PMS, Pearson correlation, trend line with 95%-CI). **F** Johnson–Neyman plot demonstrated that the association between disease duration and VD_SVC_ is age-dependent and only significant below 57.5 years of age. **G** Rank-based linear regression model was used to compare HC with PMS grouped as  ≤ 5, 6–10 and > 10 years of disease duration, including age as covariant. PMS subjects with > 10 years of disease duration had reduced VD_SVC_ compared to HC and PMS subjects with ≤ 5 years of disease duration. **H** EDSS and VD_SVC_ are negatively associated (PMS, Pearson correlation, trend line with 95%-CI). *EDSS* expanded disability status scale, *FAZ* fovea avascular zone, *HC* healthy controls, *ns* not significant (*p* ≥ 0.05), *PMS* progressive multiple sclerosis, *VD*_*DVC*_ vessel density in the deep vascular complex, *VD*_*SVC*_ vessel density in superficial vascular complex

First, we compared OCT- and OCTA-derived metrics between PMS and HC: In the PMS cohort, the pRNFL thickness (median (IQR) in μm: 94.5 (87.6–97.9)), mRNFL (mean ± SD in mm^3^: 0.85 ± 0.11) and mGCIP volumes (in mm^3^: 1.87 ± 0.18) were reduced compared to HC (99.0 (90.2–106.0), *p* = 0.024; 0.92 ± 0.10, *p* < 0.001; 2.01 ± 0.13, *p* < 0.001) (**Table 1**). The VD_SVC_ (%) was reduced in the PMS cohort compared to HC (35.4 ± 2.4 *vs* 36.6 ± 2.3, *p* = 0.016). When stratifying the VD_SVC_ by vessel diameter (%), there was a relevant reduction of vessels with a diameter > 20 μm in PMS subjects (6.4 ± 1.0 *vs* 7.0 ± 0.9, *p* = 0.002) (Fig. 2A). There was no difference between the two cohorts regarding VD_DVC_ nor the FAZ area (Table 1).

### OCTA metrics correlation with OCT-derived retinal layer metrics

Second, we explored structural associations between retinal microvasculature and neuroaxonal integrity in PMS (Fig. 2B): In PMS, the VD_SVC_ correlated with the pRNFL thickness (*β* = 0.087, SE = 0.030, *p* = 0.005), the mGCIP volume (*β* = 6.357, SE = 1.507, *p* < 0.001), respectively, the mIPL volume (*β* = 14.707, SE = 3.645, *p* < 0.001). The VD_DVC_ was associated with the mOPL volume (*β* = 9.178, SE = 2.622, *p* < 0.001).

### OCTA metrics correlation with demographic and clinical parameters

Third, we investigated the influence of demographic factors on retinal vessels: Within MS subjects, the age correlated with the VD_SVC_ (*β* = –0.095, SE = 0.031, *p* = 0.003) and the VD_DVC_ (*β* = –0.057, SE = 0.019, *p* = 0.004) (Fig. 2C, D). However, the effect of age on the VD did not differ between PMS and HC (linear regression with interaction term between age and subgroups (HC, PMS) for VD_SVC_: *β* = –0.005, SE = 0.036, *p* = 0.887; for VD_DVC_ *β* = 0.006, SE = 0.022, *p* = 0.782). In a regression model, considering age as a covariate, there was no influence of gender on VD_SVC_ nor VD_DVC_ in either the PMS or the HC cohort.

Last, we studied associations between clinical parameters and retinal VD: A multiple linear regression demonstrated that the relationship between the disease duration and the VD_SVC_ is age-dependent (*β* = 0.010, SE = 0.004, *p* = 0.029) (Fig. 2E). A Johnson–Neyman analysis identified 57.5 years as age threshold: Below this age the disease duration had a negative effect on the VD_SVC_ (Fig. 2F). In a rank-based linear regression model considering age as covariant, PwPMS with disease duration > 10 years had reduced VD_SVC_ compared to HC (*p* = 0.032) and PwPMS with ≤ 5 years of disease duration (*p* = 0.049) (Fig. 2G). Regarding age, there was no relevant association between the disease duration and the VD_DVC_ (*β* = 0.002, SE = 0.003, *p* = 0.535).

The interaction effect between age and EDSS was not significant in the overall regression model (*β* = 0.024, SE = 0.020, *p* = 0.230). The additive model showed that EDSS was negatively associated with VD_SVC_ (*β* = −0.487, SE = 0.183, *p* = 0.010) (Fig. 2H). An exploratively Johnson–Neyman analysis revealed an age range (26–56.8 years) in which EDSS was significantly negatively associated with the VD_SVC_ (supplementary figure). The EDSS did not associate with the VD_DVC_ (*β* = −0.174, SE = 0.113, *p* = 0.128).

## Discussion

This analysis examined OCTA for the first time as a tool to measure retinal microvasculature changes in a progressive MS cohort. For this purpose, we used a novel deep learning-based analysis tool to analyze OCTA images [20, 21]. To this end, we compared the OCTA-derived measurements (VD_SVC_, VD_DVC_, FAZ) of 62 PMS and 43 HC subjects who met the inclusion criteria after rigorous quality control. Overall, we detected a reduced VD_SVC_ within our PMS cohort, which on one hand correlated with OCT markers of retinal neuroaxonal loss, and on the other hand associated with disease duration and disability mainly in younger subjects.

The characteristics of our cohorts align with previously reported OCT data, indicating that the study population is representative and appropriate for evaluating the potential of OCTA: Reduced values of pRNFL thickness and mGCIP volume were observed. According to the literature, the reduced retinal thickness is associated with the clinical course of the disease and with magnetic resonance imaging-based (MRI) markers of neurodegeneration [22–24]. However, (asymptomatic) optic nerve lesions as well as lesions within the postchiasmatic visual pathway might explain reduced retinal thickness in our PMS cohort [25]. The absence of MRI data constitutes a limitation of the present analysis. To address this, future OCTA studies should incorporate multimodal MR imaging. The reduced mRNFL volume described here is less well-studied in MS but has also been repeatedly reported [10, 26]. In our cohort, there was no difference in mINL between PMS and HC.

We reported a reduced VD_SVC_ in PMS, while there were no relevant differences in the VD_DVC_ and FAZ area compared to HC. These results are in line with a meta-analysis of OCTA data in predominantly RMS: Here, a relevant reduction in VD_SVC_ was reported, even across different OCTA devices, and even when only eyes of MS subjects without ON were compared with HC [11]. In recent studies using the same OCT/OCTA device as our analysis, neither the VD_DVC_ nor the FAZ area was described as a relevant target structure in a cross-sectional or longitudinal analysis of PwRMS [12, 13]. Furthermore, we demonstrated that retinal VD correlates with corresponding retinal layer volumes. Similar results have recently been shown in RMS [27, 28]. However, it is currently pathophysiologically unclear whether a pathology of small vessels is a driver of neurodegenerative processes in MS, potentially leading to (secondary) reduced retinal layer thickness [29]. Or whether—as possibly explained by histopathological examinations of retinas in PwMS—the comparatively low loss of retinal vascular density in PPMS is rather itself a secondary consequence following pronounced ganglion cell loss [30]. In summary, our results discussed so far reflect important trends in OCTA in RMS for the first time in an exclusively PMS cohort. However, external validation and pathophysiological understanding of the observed effects are pending.

It has been repeatedly shown that VD is age-dependent in healthy eyes [31, 32]. Our results further support that age should always be considered when evaluating OCTA in PMS. Longitudinal studies are needed to further investigate whether age-related processes might differ between HC and PMS individuals. We described a relevant interaction between age and disease duration, negatively correlating with VD_SVC_. In addition, our analysis showed a correlation between clinical disability and VD_SVC_. Although the interaction effect (EDSS: age) was not significant in the overall model, an exploratory Johnson–Neyman analysis provides indication of age-dependent heterogeneity in the relationship between EDSS and VD_SVC_. Consistent to the assessment of disease duration, this analysis suggests that disability is more strongly associated with VD_SVC_, particularly in PMS subjects under 56.8 years of age. So far, similar correlations between disease duration or functional score and retinal VD have only been shown for persons with clinically isolated syndrome or RMS, i.e., particularly in the context of inflammatory disease stage [28, 33]. Based on our data, we hypothesize that the strength of OCTA could be the detection of changes in the retinal microvasculature, particularly in younger PMS individuals with longer disease duration. In contrast, in older PMS individuals with a short disease duration, it is likely difficult to distinguish MS-related effects on retinal VD from the effects of age (and comorbidities). Taken together, we consider the possibilities of OCTA to detect early neurodegenerative processes in PMS to be limited. Further studies with larger cohorts are needed to validate and mechanistically understand these possible associations.

This study is limited mainly due to its cross-sectional nature and cohort size. Furthermore, OCTA is associated with longer scan time, more challenging image acquisition and a higher level of compliance is required from the examined individual compared to OCT. In line with this, it was shown that OCTA artifacts are more common in MS than in HC individuals [34]. As a result, severely impaired individuals might be under-represented in OCTA analyses, which significantly limits the role of OCTA in MS. A substantial proportion of OCTA images was not eligible for our analysis. This likely reflects intrinsic challenges of OCTA acquisition in clinical populations, including increased artifact susceptibility in more advanced disease stages, while comparative benchmarks for image eligibility rates across centers are largely unavailable. Furthermore, the extensibility of OCTA is currently limited due to methodological heterogeneity (OCTA device, analysis tool) [35]. Longitudinal OCTA analyses with larger cohorts are needed to assess if OCTA holds further potential as a complementary tool for monitoring disease progression in MS. These analyses should consider combining OCTA metrics with other methods for evaluating disease progression in MS, e.g., MRI and blood or cerebrospinal fluid-based biomarkers [1].

In summary, we observed vessel rarefication mainly in the SVC using OCTA in a purely progressive MS cohort. This effect was particularly pronounced in younger individuals with longer disease duration. External validation and longitudinal studies on the use of OCTA in PMS are pending.

## Supplementary Information

Below is the link to the electronic supplementary material.

Supplementary file1 (DOCX 46 KB)

## Data Availability

Data displayed are only available for justified requests.
