# Supplementary material for: The potential of optical coherence tomography angiography in progressive multiple sclerosis
Source: J Neurol. 2026 Feb 7;273(2):118. doi: 10.1007/s00415-026-13659-7 (PMC12882947; doi:10.1007/s00415-026-13659-7)
Supplement: Supplementary file 1 — Supplementary file1 (DOCX 46 KB) [file 415_2026_13659_MOESM1_ESM.docx]

# Supplementary Fig.


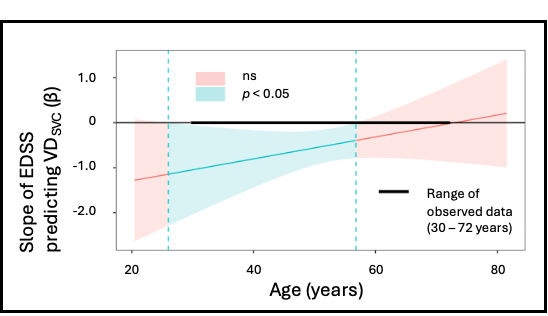


Supplementary Fig. Explorative Johnson–Neyman analysis

An exploratory Johnson–Neyman analysis was performed—as the interaction term for the relationship between age and EDSS was not significant in the regression model. Comparable to the trend regarding the influence of disease duration on VD_SVC_ (vessel density in the superficial vascular complex) (taking age into account), the present analysis suggests a critical interval between 26 and 56.8 years, where the EDSS negatively correlates with the VD_SVC_.
